# Functional fusion of living systems with synthetic electrode interfaces

**DOI:** 10.3762/bjnano.7.27

**Published:** 2016-02-26

**Authors:** Oskar Staufer, Sebastian Weber, C Peter Bengtson, Hilmar Bading, Joachim P Spatz, Amin Rustom

**Affiliations:** 1 Max-Planck Institute for Intelligent Systems, Department of New Materials and Biosystems, Heisenbergstraße 3, D-70569 Stuttgart, Germany; 2 German Cancer Research Center, DKFZ Life Science Lab, Im Neuenheimer Feld 581, D-69120 Heidelberg, Germany; 3 Bachelor Program Molecular Biotechnology, University of Heidelberg, Institute of Pharmacy and Molecular Biotechnology, Im Neuenheimer Feld 364, D-69120 Heidelberg, Germany; 4 Department of Neurobiology, Interdisciplinary Centre for Neurosciences (IZN), University of Heidelberg, Im Neuenheimer Feld 364, D-69120 Heidelberg, Germany,; 5 University of Heidelberg, Department of Biophysical Chemistry, Im Neuenheimer Feld 253, D-69120 Heidelberg, Germany

**Keywords:** biointerface, biosensor, energy harvesting, nanoelectrodes, *Physarum polycephalum*

## Abstract

The functional fusion of “living” biomaterial (such as cells) with synthetic systems has developed into a principal ambition for various scientific disciplines. In particular, emerging fields such as bionics and nanomedicine integrate advanced nanomaterials with biomolecules, cells and organisms in order to develop novel strategies for applications, including energy production or real-time diagnostics utilizing biomolecular machineries “perfected” during billion years of evolution. To date, hardware–wetware interfaces that sample or modulate bioelectric potentials, such as neuroprostheses or implantable energy harvesters, are mostly based on microelectrodes brought into the closest possible contact with the targeted cells. Recently, the possibility of using electrochemical gradients of the inner ear for technical applications was demonstrated using implanted electrodes, where 1.12 nW of electrical power was harvested from the guinea pig endocochlear potential for up to 5 h (Mercier, P.; Lysaght, A.; Bandyopadhyay, S.; Chandrakasan, A.; Stankovic, K. *Nat. Biotech.* **2012,** *30,* 1240–1243). More recent approaches employ nanowires (NWs) able to penetrate the cellular membrane and to record extra- and intracellular electrical signals, in some cases with subcellular resolution (Spira, M.; Hai, A. *Nat. Nano.* **2013,** *8,* 83–94). Such techniques include nanoelectric scaffolds containing free-standing silicon NWs (Robinson, J. T.; Jorgolli, M.; Shalek, A. K.; Yoon, M. H.; Gertner, R. S.; Park, H. *Nat Nanotechnol.* **2012,** *10,* 180–184) or NW field-effect transistors (Qing, Q.; Jiang, Z.; Xu, L.; Gao, R.; Mai, L.; Lieber, C. *Nat. Nano.* **2013,** *9,* 142–147), vertically aligned gallium phosphide NWs (Hällström, W.; Mårtensson, T.; Prinz, C.; Gustavsson, P.; Montelius, L.; Samuelson, L.; Kanje, M. *Nano Lett.* **2007,** *7,* 2960–2965) or individually contacted, electrically active carbon nanofibers. The latter of these approaches is capable of recording electrical responses from oxidative events occurring in intercellular regions of neuronal cultures (Zhang, D.; Rand, E.; Marsh, M.; Andrews, R.; Lee, K.; Meyyappan, M.; Koehne, J. *Mol. Neurobiol.* **2013,** *48,* 380–385). Employing monocrystalline gold, nanoelectrode interfaces, we have now achieved stable, functional access to the electrochemical machinery of individual *Physarum polycephalum* slime mold cells. We demonstrate the “symbionic” union, allowing for electrophysiological measurements, functioning as autonomous sensors and capable of producing nanowatts of electric power. This represents a further step towards the future development of groundbreaking, cell-based technologies, such as bionic sensory systems or miniaturized energy sources to power various devices, or even “intelligent implants”, constantly refueled by their surrounding nutrients.

## Findings

**Figure 1:**
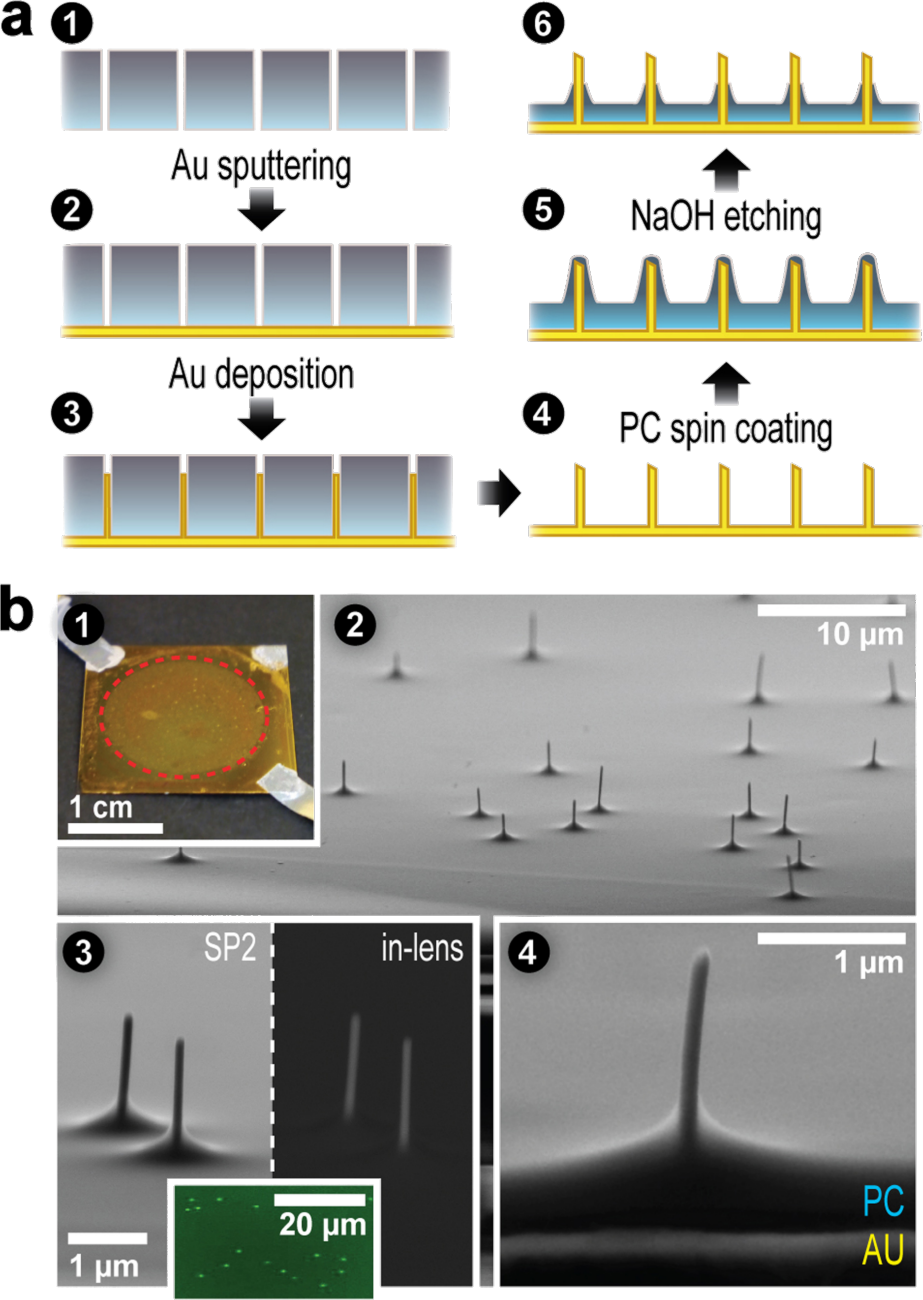
(a) Fabrication of nanoelectrode interfaces (NEIs). Track-etched, polycarbonate (PC) filter membranes (a, 1) are sputter-coated with a gold layer and applied to titanium/gold coated coverslips (a, 2). During wet chemical electrodeposition, monocrystalline gold pillars grow within the filter pores (a, 3). After dissolving the filter membrane using dichloromethane (DCM), free-standing electrodes (a, 4) are covered with a layer of PC via spin-coating (a, 5). Brief etching with NaOH finally exposes the electrode tips (a, 6). (b) Ultrastructural analysis of NEIs. A photograph of a final NEI including the attached Al contacts is shown in (b, 1). SEM analysis of the respective surfaces shows that the 1.57 cm^2^ deposited area (red dotted line) is covered with partly isolated gold electrodes (b, 2). The electrodes have an average diameter of 102 ± 12 nm and length of 2 ± 0.3 µm (b, 3). Corresponding images with the in-lens detector provide increased material contrast to highlight the nonisolated electrode tips (b, 3, right). The accessibility of the gold surface is proven by fluorescence microscopy analysis of NEIs in which gold was labeled by a biotin- and AlexaFluor488-labeled streptavidin (b, 3, inset). A SEM side view obtained at a centric braking edge reveals the composition of the interface illustrating gold pillars protruding from the isolating PC layer (b, 4).

The formation process of nanoelectrode interfaces (NEIs) was based on track-etch template synthesis as schematically shown in Figure 1a. First, monocrystalline gold (Au) nanowires in parallel arrangement featuring homogenous, low defect and parallel-oriented crystal planes were produced by electrodeposition as previously described [1]. This process employed polycarbonate (PC), track-etched filter membranes with a pore density of 1 × 10^6^ pores/cm^2^ and a pore size of 100 nm (Figure 1a, 1–4). Aluminium (Al) contacts were attached to the surface with a conductive silver lacquer. An isolating, PC layer was applied by spin coating at 1500 rpm (Figure 1a, 5). Finally, the surfaces were etched with 2 N NaOH for 12 min at 70 °C in order to selectively expose the electrode tips (Figure 1a, 6).

**Figure 2:**
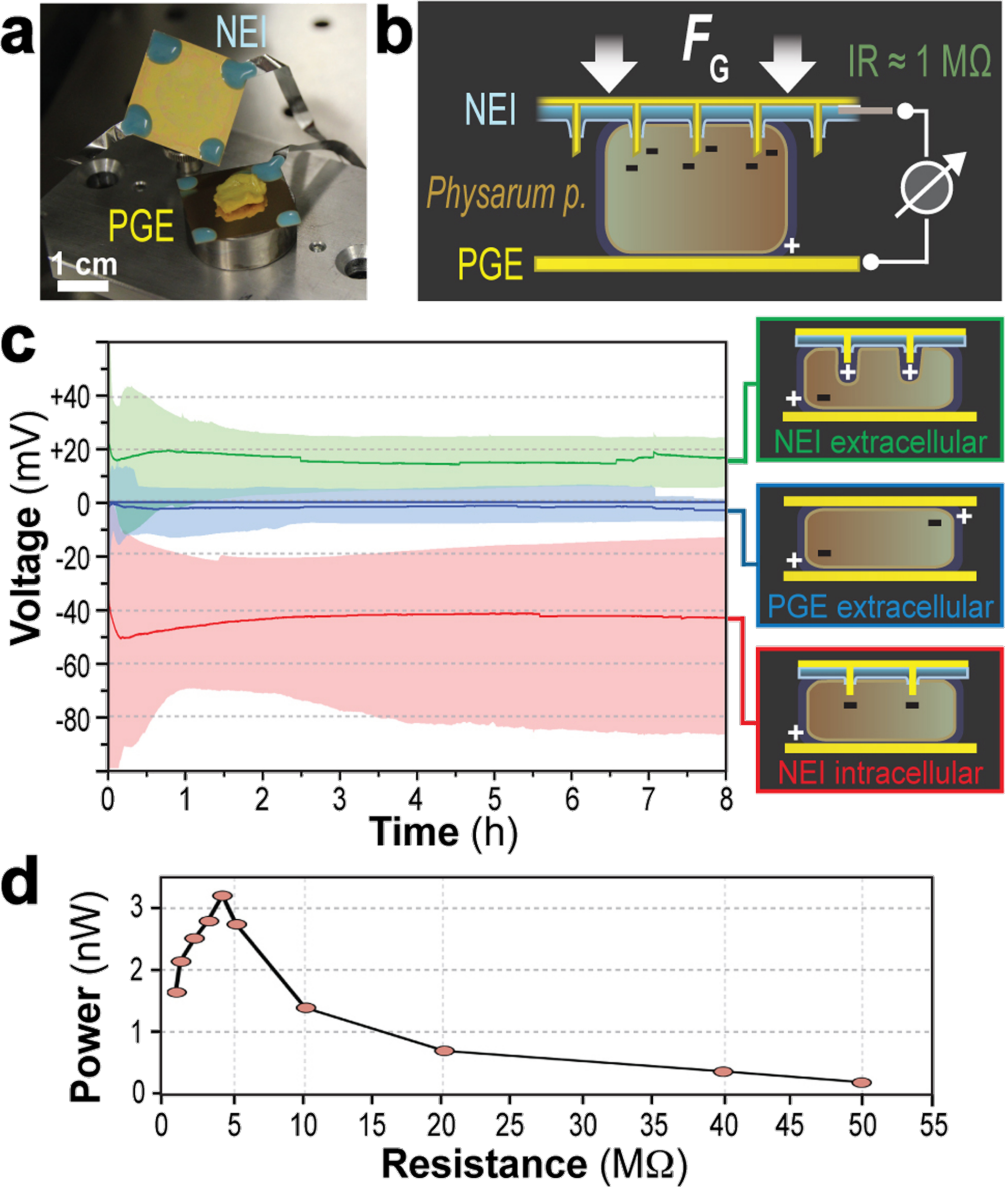
“Symbionic” slime mold/NEI unions. A fragment of *Physarum p.* was placed between a PGE and an NEI (a) and a weight of 5 g was placed on top (*F*_G_) to promote uniform electrode penetration (b). The resulting voltage was monitored for at least 8 h with an oscilloscope-based setup with an input resistance of 1 MΩ (c). Traces show the average (line) and range (shaded area) of recordings from *Physarum p.* sandwiched between either two PGEs or a PGE and an NEI. PGE recordings (blue) represent the extracellular potential (on average zero). NEI/PGE recordings showed either slightly positive voltages (green), presumably reflecting the extracellular potential with a NEI/PGE local extracellular potential, or negative voltages (red), presumably reflecting intracellular, nanoelectrode localization with a −40 mV membrane potential. For each condition, the mean of *n* = 10 independent experiments is shown. Maximal electric output of the intracellularly connected *Physarum*/NEI/PGE configuration was determined by measurements using defined resistors (d).

**Figure 3:**
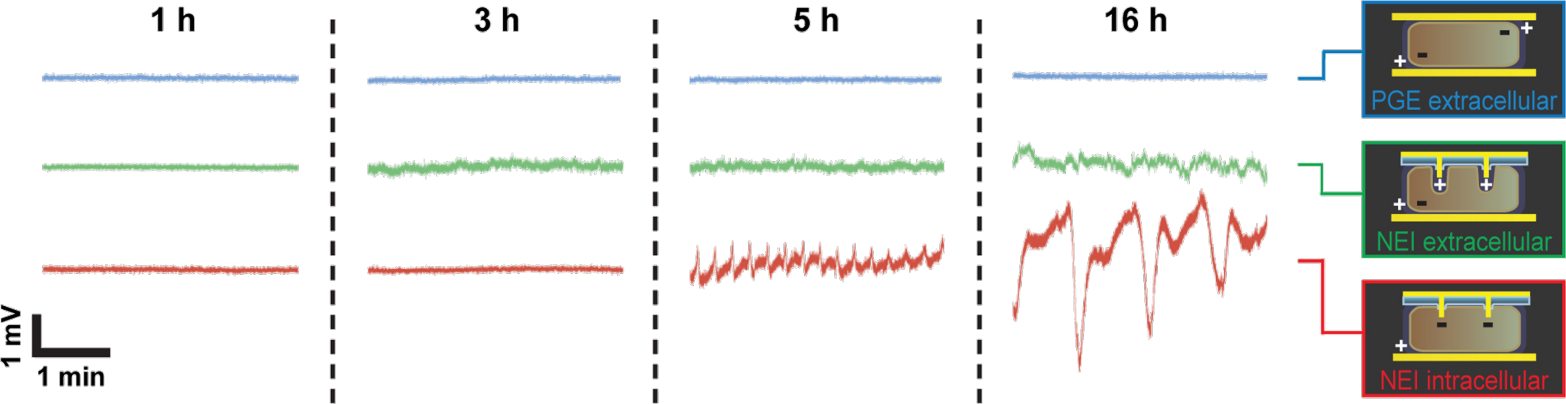
Membrane potential oscillations of *Physarum p.* measured with NEIs. Typical recordings from *Physarum p.* sandwiched between NEI/PGE or PGE/PGE surfaces are shown at the indicated time points after connecting the circuit to the electrical load. Membrane potential oscillations, presumably associated with the protoplasmic streaming activity of *Physarum p.*, were frequently detected after 3 h in the case of extracellular NEIs and (albeit less frequently) after 5 h in the intracellular configuration. Note that the signal amplitude has strongly increased in the later case. Representative recordings are shown.

**Figure 4:**
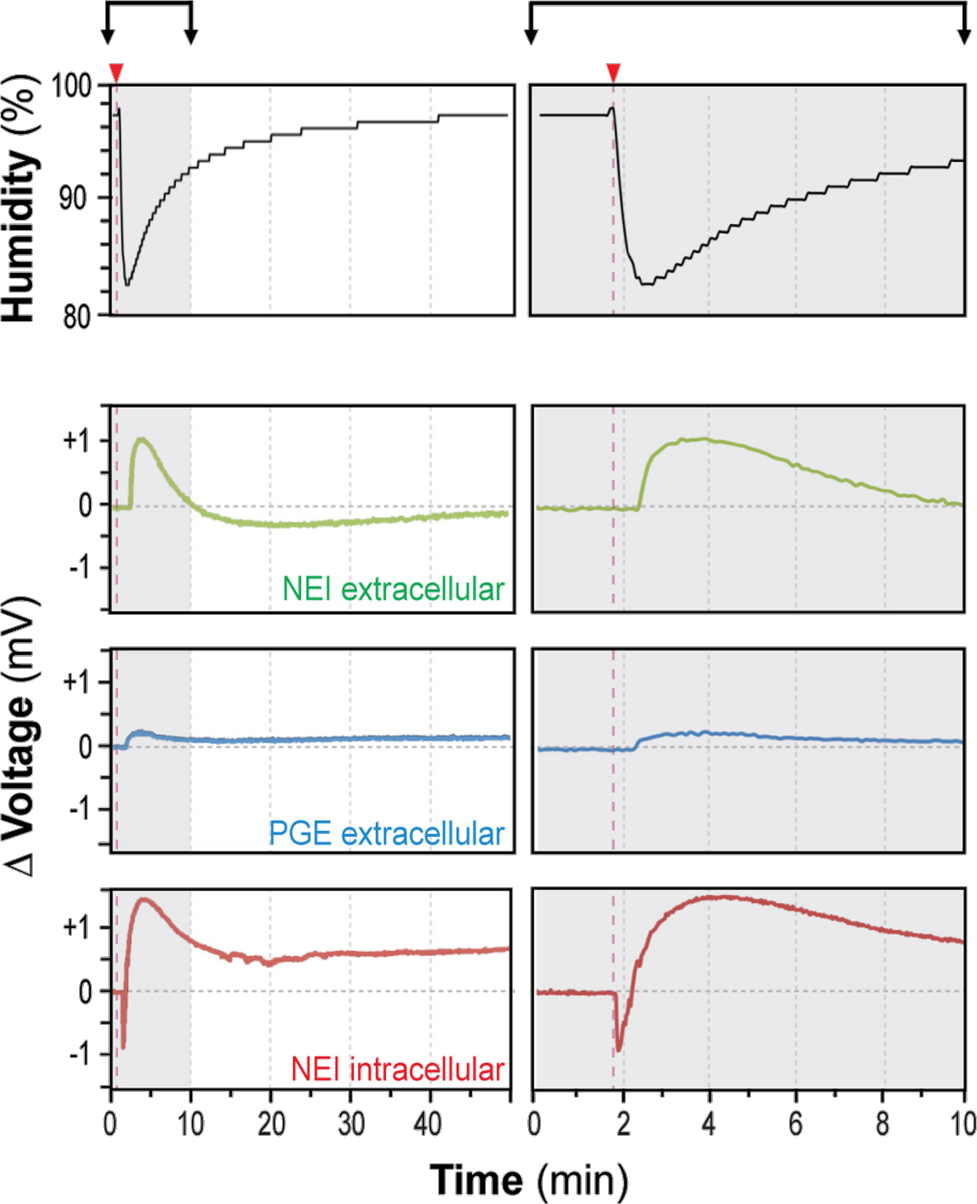
*Physarum p.*-based, autonomous humidity sensing. *Physarum p.* sandwiched between NEI/PGE or PGE/PGE surfaces was exposed to changes in incubator humidity (black) using a custom-built ventilation system (see Supporting Information File 1). Briefly, transient drops in humidity evoked selectively in NEI/PGE electrodes with negative potentials (indicative of intracellular, nanoelectrode localization) a biphasic response (red), consisting of a short hyperpolarization and longer depolarization phase. PGEs (blue) or “extracellular” NEI/PGEs (green) showed instead a delayed monophasic response to transient decreases in humidity. Representative recordings are shown.

Scanning electron microscopy (SEM) analysis of the final NEI surface (Figure 1b, 1) shows the deposited circular area of 1.57 cm^2^ on the glass coverslip covered with 1 × 10^6^ electrodes/cm^2^ with an average length of 2 ± 0.3 µm and diameter of 102 ± 12 nm (Figure 1b, 2). The laid open electrode tips can be highlighted by use of an in-lens SEM detector enhancing material contrast (Figure 1b, 3) and accessibility of the gold surface demonstrated by specific labelling with a biotin and AlexaFluor 488 labelled streptavidin, resulting in punctured fluorescence signals at the expected density (Figure 1b, 3, inset). By use of a profilometer (see Supporting Information File 1, Experimental) and comparing the length of isolated and nonisolated electrodes in SEM cross sections the thickness of the PC layer was determined to be 405 ± 5 nm (Figure 1b, 4). All surface parameters had to be thoroughly tuned in order to achieve a balance between penetration ability, isolation properties and cell survival on one hand, and mechanical stability on the other.

To demonstrate the ability of our NEIs to mediate functional access to the interior of living cells, we fused them with macroplasmodia of the slime mold *Physarum polycephalum* (Figure 2). This developmental stage represents an individual multinucleated cell confined by one continuous plasma membrane, featuring complex membrane potential oscillations [2] correlated with problem solving strategies [3–4] and electrochemical reactions to varying environmental conditions, including light [5] and humidity [6]. Along with its robustness and ease of cultivation (see Supporting Information File 1), these properties make *Physarum p.* an ideal model organism for various bioinspired technologies. To plate the slime mold onto NEIs, approximately 400 µL of a *Physarum p.* confluent advancing front was scraped with a spatula from a 2–3 day-old macroplasmodium and placed upside down onto a planar gold electrode (PGE) mounted on a nonconductive holding platform (Figure 2a,b). As *Physarum p.* exhibits rapid membrane regeneration after rupture, this macroplasmodial mass was allowed to recover for 1–2 min before mounting the NEI on top of the assembly. For additional pressure, a 5 g weight was placed on the setup (Figure 2b, *F*_G_) to enforce membrane penetration and overcome the low proportion and stochastic nature of nanoelectrode penetration observed for mammalian CHO cells using hollow alumina nanostraws [7]. The electrodes were short-circuited before each measurement to prevent further artefacts, such as remaining surface charges. Voltage measurements were performed using an oscilloscope-based setup with an input resistance of 1 MΩ to simulate an electrical load on the circuit (see Supporting Information File 1).

The measurements from *Physarum p.* sandwiched between a PGE and an NEI resulted in two distinctive states. In a first set of experiments, a mean voltage of −40 ± 6 mV was detected (Figure 2c, red), corresponding to published estimates of membrane potential of *Physarum p.* plasmodia, ranging from −20 to −100 mV [8–9]. This was also in line with our comparative measurements performed by conventional single glass pipette-based electrophysiology (see Supporting Information File 1), which detected −35 ± 12 mV (Supporting Information File 1, Figure S1a). Noteworthy, the potential measured with our NEI/PGE system was stable in most cases for the complete observation period, which lasted several days and sometimes up to one week (Supporting Information File 1, Figure S1b). In a few cases, the potential dropped more or less slowly, most likely reflecting successive ejection of nanoelectrodes from the cytoplasm or, at later time points, nutrient shortage. This view is corroborated by our own (Supporting Information File 1, Figure S1a) as well as other various electrophysiology studies, observing rapid ejection of intracellular glass microelectrodes from *Physarum p.* [10]. This is presumably due to its pronounced membrane regenerative ability. In line with this notion, the observed voltage drop was accelerated when NEI measurements were performed without additional pressure applied to the electrode (Supporting Information File 1, Figure S1c). Considering the input resistance of the system, −40 mV corresponds to a hypothetical electrical output of approximately 1.6 nW. To determine the maximal electrical output of this *Physarum*/NEI/PGE “battery”, measurements using defined resistors were performed. This experiment revealed a peak of 3.31 nW at 4 MΩ (Figure 2d), emphasizing its potential use for energy harvesting applications.

In the second set of experiments, a mean voltage of +20 ± 8 mV was detectable (Figure 2c, green). This is interpreted as NEIs not being able to penetrate the plasma membrane, resulting in an “extracellular” configuration. An explanation for the volatile penetration ability could be minor alterations of NEI surface quality based on the manual production process and/or variances of the slime mold constitution (e.g., the thickness of the slime layer). This combination of nanostructural and biological variability is difficult to control due to the complexity of the experimental setup. Indeed, several cell/NW-related papers based on experimental and theoretical considerations including mathematical and mechanical models predict a narrow window for the aspect ratio and density of electrodes as well as for factors such as cell stiffness, cell spreading, substrate adhesion or cell traction forces in order to ensure efficient membrane penetration [11–12]. Extracellular control measurements performed with PGE/PGE instead of NEI/PGE configurations revealed a stable voltage of only −0.5 ± 2 mV (Figure 2c, blue**)**.

Interestingly, during “extracellular” NEI measurements, sinusoidal potential changes frequently became detectable (Figure 3, green). These are well understood for *Physarum p.* and closely related to its oscillating cytoplasmic shuttle streaming that develops over a similar time frame [2]. When compared to PGE/PGE recordings, the significant enhancement of the signal-to-noise ratio is likely due to an increased electrical coupling coefficient based on the “close” contact between the sharp, monocrystalline electrode tips and the plasma membrane in combination with the PC isolation layer increasing the seal resistance. Comparable improvements of the signal-to-noise ratio were achieved with several nano- or microstructured electrode surfaces, such as chemically functionalized micrometer-sized mushroom-shaped gold protrusions [13–14], highlighting the general advantages of such surfaces over planar electrodes. The signal-to-noise ratio of intracellular NEI measurements (Figure 3, red) was even better, presumably due to their direct, low impedance access to the cell interior, proving the suitability of the system for electrophysiological recordings and biosensor applications. The fact that under these conditions the periodic potential changes were only rarely observed (and if so, at later time points) could mean that the artificial electrical access has significant impact on the underlying electrochemical machinery. This finding may be used in the future to specifically modulate cellular behavior by input of defined electrical signals.

The successful demonstration of stable electrical access to the membrane potential of cells opens the door for various applications requiring direct or indirect access to the electrochemical machinery of biological structures. To demonstrate the capabilities of our NEI technology in view of typical sensor applications, we used the *Physarum*/NEI preparation as an “autonomous” humidity sensor, employing the cell’s intrinsic capability to respond to environmental humidity changes [6]. *Physarum*/NEI sandwiches were prepared as described and the potential was recorded over time (Figure 4) while the humidity inside the experimental chamber was varied using a custom-built ventilation system (see Supporting Information File 1). An abrupt reduction in humidity resulted in a biphasic electrical response, consisting of a rapid hyperpolarization in the range of −1 mV followed by a slower depolarization of approximately +1.5 mV (Figure 4, red). Noteworthy, the amplitude of this response was dependent on the strength of the humidity reduction, proven by experiments employing different humidity levels (Supporting Information File 1, Figure S1d).

In contrast, extracellular measurements employing PGEs showed a delayed, monophasic deflection in the range of only +0.2 mV (Figure 4, blue). “Extracellular”, NEI-based measurements showed the same monophasic progression but with a larger amplitude in the range of +1 mV (Figure 4, green). Although the cellular mechanisms behind hygrosensing of *Physarum p.* are largely unknown (as is true for hygroreceptors from various insects [15–16]), our observations demonstrate that even complex cell functions may be technologically implemented by an appropriate interface and interpretation of their characteristic signal patterns. The remarkable delay in the appearance of the monophasic electrical response due to humidity changes in our “extracellular” measurements was most likely related to the lack of a hyperpolarization phase. This effect may relate to the participation of intracellular Ca^2+^ stores, as previously suggested with regard to ATP/P2x purinergic receptor-induced potential changes [17].

In summary, we have demonstrated functional access to the electrochemical machinery of a eukaryotic cell system that may open the door to innovative sensor and energy harvesting applications. By employing a cell’s intrinsic signal processing and amplifying strategies, based on receptors, ion channels and signaling cascades, future applications of these technologies may attain molecular precision and/or improved energy conversion efficiency. Furthermore, since NEI analysis is not limited to single or small numbers of cells, it also allows for electrophysiological measurements and potential recordings from complete cell layers. This could compensate for individual variation and serve to amplify weak electric output for improved detection of functions such as intercellular communication.

## Supporting Information

File 1 Additional experimental information.
