# Supplementary material for: Functional fusion of living systems with synthetic electrode interfaces
Source: Beilstein J Nanotechnol. 2016 Feb 26;7:296–301. doi: 10.3762/bjnano.7.27 (PMC4778514; doi:10.3762/bjnano.7.27)
Supplement: File 1 — Additional experimental information. [file Beilstein_J_Nanotechnol-07-296-s001.pdf]

Supporting Information

for

Functional fusion of living systems with synthetic electrode interfaces

Oskar Staufer[1,2,3], Sebastian Weber[1], C. Peter Bengtson[4], Hilmar Bading[4], Joachim P. Spatz[1,5] and Amin Rustom*[1,5]

Address: [1]Max-Planck Institute for Intelligent Systems, Department of New Materials and Biosystems, Heisenbergstraße 3, D-70569 Stuttgart, Germany, [2]German Cancer Research Center, DKFZ Life Science Lab, Im Neuenheimer Feld 581, D-69120 Heidelberg, Germany, [3]Bachelor Program Molecular Biotechnology, University of Heidelberg, Institute of Pharmacy and Molecular Biotechnology, Im Neuenheimer Feld 364, D-69120 Heidelberg, Germany, [4]Department of Neurobiology, Interdisciplinary Centre for Neurosciences (IZN), University of Heidelberg, Im Neuenheimer Feld 364, D-69120 Heidelberg, Germany, and [5]University of Heidelberg, Department of Biophysical Chemistry, Im Neuenheimer Feld 253, D-69120 Heidelberg, Germany

Email: Amin Rustom - amin.rustom@urz.uni-heidelberg.de

* Corresponding author

Additional experimental information

Experimental

NEI fabrication

NEIs were fabricated as described before (Schneckenburger, M.; Kelsch, M.; van Aken, P.; Richter, G.; Spatz, JP.; Rustom, A. *Small* **2012**, 8, 3396-3399). Briefly, 24 x 24 mm glass cover slips were cleaned from organic residues by a 1 h incubation in peroxymonosulfuric acid, followed by a ultrasonic cleaning in distilled water and subsequently dried by a nitrogen stream. They were covered with a 50 nm thick Au layer sputtered on top of a 5 nm supporting Ti layer (Multi-Coating-System MCS 010, Bal-Tec GmbH, Witten). For NEIs 25 μm thick track-etched PC membranes (it4ip, Belgium), with pore diameters of 100 nm and a pore density of 10^6 /cm^2 were covered with a 25 nm thick Au layer, placed upside down onto the prepared cover slips and mounted onto a custom-built deposition system. Electrochemical deposition was performed with gold electrolyte (Conrad Electronics SE, Hirschau) for 900 s at 1.5 V resulting in a mean electrode length of 2 μm. PC membranes were removed by an overnight dichloromethane (DCM) (Merck KGaA, Darmstadt) incubation followed by a 15 min H_2O wash. Nanoelectrodes were isolated by a PC layer (polybisphenol-A-carbonate, Sigma-Aldrich GmbH, Taufkirchen) applied over a spin coating process (Multi-Coating-System MCS 010, Bal-Tec GmbH, Witten) and dried at 65 °C for 3 min. To selectively uncover the electrode tips, a 2N NaOH solution was applied to the surfaces at 70 °C for 12 min and subsequently washed away. Prior to voltage measurements, electrode surfaces were coated with poly-L-lysine (Sigma-Aldrich

GmbH, Taufkirchen) to decrease surface hydrophobicity and to enhance cell / NEI contact formation.

Scanning Electron Microscopy (SEM) analysis

NEIs were analysed by SEM by covering surfaces with a carbon layer. Samples were imaged with a ZEISS LEO 1530 in-lens field emission scanning electron microscope (Carl Zeiss NTS GmbH, Oberkochen, Germany).

Culture of *Physarum p.*

Physarum p. was routinely cultured axenically in the macroplasmodial stage on nutrient agar plates according to the methods of Rush [Daniel, W.; Rush, H. *Microbiology* **1961**, 25, 47-59]. Briefly, 2% agar plates were poured with a balanced salt solution containing yeast extract, glucose, tryptone and 1% hemin adjusted to pH 4.6. Macroplasmodia were grown in the dark at 25 °C and 100% humidity. Subcultures were made every 3-4 days by transferring a small piece of macroplasmodium with its supporting agar onto a new nutrient agar plate. Strain IK1 used in this study was developed by crossing amoebeal strains LU897 (Anderson, Truitt) and LU352 (Dee, Foxon, Anderson), obtained from Prof. Wolfang Marwan (University of Magdeburg), as has been detailed elsewhere [Anderson, R. *Genetics* **1979**, 91, 409 - 419].

NEI measurements

All measurements were performed in an Eppendorf Galaxy 14S incubator (New Brunswick Scientific, USA) at room temperature (22 °C). Temperature and humidity were recorded with a Lascar EL-USB-1 logger inside the incubator (10 s logging

rate). Humidity was varied by pumping the air inside the incubator through 250 g dry silica gel beads with an Evolution Silent Mouse M-106 air pump (120 L/h flow rate) situated outside of the incubator. Voltage recordings were performed with a MEphisto UM202 oscilloscope (Meilhaus Electronics, Puchheim) controlled by a LabView software (National Instruments Corporation, USA).

Single electrode measurements

Recordings were made on a fixed-stage upright microscope (BX51WI, Olympus) using a Multiclamp 700 A amplifier, digitized through a Digidata 1322A A/D converter and acquired and analyzed using pClamp 10 software (Molecular Devices). Electrodes (2–3 MΩ) were made from 1.5 mm borosilicate glass and filled with internal solution (in mM: KCl, 20; NaCl, 10; $CaCl_2$, 3; EGTA, 10; PIPES, 15; Mg_2ATP, 25; glucose, 30; pH 6 with KOH, osmolarity 150mOsm).

Staining of exposed gold surfaces

In a closed glass chamber, samples were placed on teflon holders standing in an ethanol bath for ethanol saturation. Up to 200 µL biotin (Sigma-Aldrich GmbH, Taufkirchen) working solution were applied to each sample, held by a Twinsil® rim. After incubating for 3 h, samples were gradually washed with a mixture of EtOH and PBS starting from pure EtOH. AlexaFluor® 488 fluorescent labelled streptavidin (Sigma-Aldrich GmbH, Taufkirchen) in PBS was applied to each sample and incubated for 1 h in the dark. To remove excess stain, the samples were washed with PBS.

Profilometer measurements

Thickness of the PC-layer was measured with a Dektak Profilometer. The PC-layer was mechanically scratched with a sharp blade prior to measurement. For each spin speed three samples were measured at five different locations. Measured thickness was corrected by 55 nm accounting for the Ti/Au layer removed by scratching.

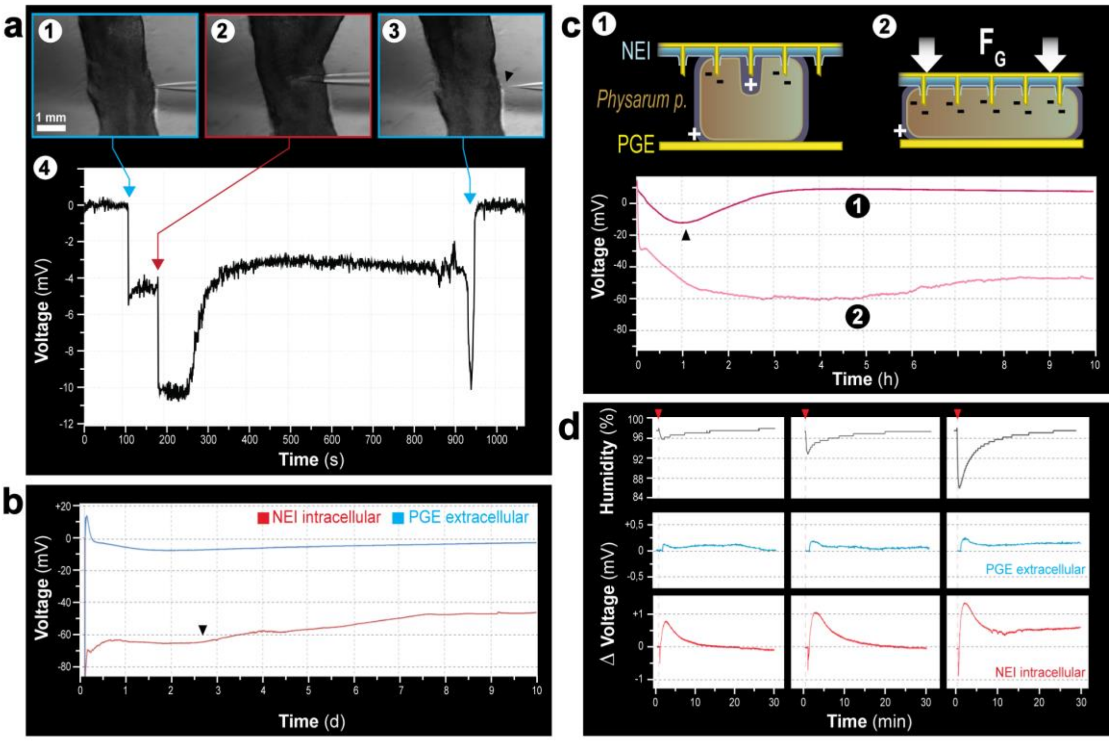

Figure S1: (a) The membrane potential of *Physarum p*. analysed by conventional single electrode electrophysiology. A 3–4 MΩ glass pipette was slowly advanced toward a protoplasmic tube until contact formation **(a1)**, resulting in a voltage drop **(a4)**. The pipette was rapidly moved forward to penetrate the membrane **(a2)**, resulting in a second voltage drop, reflecting the slime moulds membrane potential **(a4, red)**. These recordings were stable for only short time periods, often less than a minute, before the pipette potential suddenly returned to the contact value, most likely due to rapid ejection of electrodes from the cytoplasm. When the electrode was slowly

retracted from the cytoplasm, a thin tether, presumably membrane, was pulled out from the protoplasmic tube **(a3)** which upon rupture caused a short voltage peak, before returning to the non-contact value **(a4)**. (b) Long term measurement with an NEI / *Physarum p.* union. A *Physarum p.* / NEI union as well as a control setup with PGEs were prepared as described (compare to Figure 2) and analysed for 10 days. Please note that the membrane potential of the slime mould was stable for approximately 2.5–3 days. Afterwards (arrowhead) the voltage drops slowly but continuously – in this case presumably provoked by nutrient shortage rather then electrode ejection, which happens much faster (compare **c1**, arrowhead). (c) Pressure dependence of stable membrane penetration. *Physarum p.* / NEI unions were prepared as described. One was analysed without **(c1),** the other one with the 5 g weight placed on top of the NEI (**c2**). Please note that during measurements without additional weight, the voltage - after a peak at one hour (arrowhead) - rapidly declines (**c1**), indicative of electrodes being ejected from the cytoplasm. In contrast, with additional pressure, the voltage increases and stays stable for extended time periods **(c2)**, suggesting that the additional weight prevents electrodes from being ejected from the cytoplasm. (d) Quantitative aspects of *Physarum p.* based humidity sensing. *Physarum p.* / NEI or PGE unions were prepared and connected as described. The humidity inside the experimental chamber was controlled and monitored (**black**) by a custom-built ventilation system. Note that the amplitude of the slime mould response measured intracellularly (**red**), consisting of a short hyperpolarisation and longer depolarisation phase, depends on the degree of humidity reduction.
